# Quantification of Pathologic Air Trapping in Lung Transplant Patients Using CT Density Mapping: Comparison with Other CT Air Trapping Measures

**DOI:** 10.1371/journal.pone.0139102

**Published:** 2015-10-02

**Authors:** Olga Solyanik, Patrick Hollmann, Sabine Dettmer, Till Kaireit, Cornelia Schaefer-Prokop, Frank Wacker, Jens Vogel-Claussen, Hoen-oh Shin

**Affiliations:** 1 Institute of Diagnostic and Interventional Radiology, Hannover Medical School, Hanover, Germany; 2 Institute of Diagnostic and Interventional Radiology, Kantonsspital, Aarau, Switzerland; 3 Radiologie, Meander Medisch Centrum, Amersfoort, the Netherlands; 4 Radiologie – DIAG, UMC St Radboud, Nijmegen, the Netherlands; 5 Research in Endstage and Obstructive Lung Disease Hannover (BREATH), Member of the German Center for Lung Research, Hanover, Germany; University of California San Francisco, UNITED STATES

## Abstract

To determine whether density mapping (DM) is more accurate for detection and quantification of pathologic air trapping (pAT) in patients after lung transplantation compared to other CT air trapping measures. One-hundred forty-seven lung and heart-lung transplant recipients underwent CT-examinations at functional residual capacity (FRC) and total lung capacity (TLC) and PFT six months after lung transplantation. Quantification of air trapping was performed with the threshold-based method in expiration (EXP), density mapping (DM) and the expiratory to inspiratory ratio of the mean lung density (E/I-ratio MLD). A non-rigid registration of inspiration-expiration CT-data with a following voxel-to-voxel mapping was carried out for DM. Systematic variation of attenuation ranges was performed for EXP and DM and correlated with the ratio of residual volume to total lung capacity (RV/TLC) by Spearman rank correlation test. AT was considered pathologic if RV/TLC was above the 95^th^ percentile of the predicted upper limit of normal values. Receiver operating characteristic (ROC) analysis was performed. The optimal attenuation range for the EXP method was from -790 HU to -950 HU (EXP_-790 to -950HU_ *)* (*r* = 0.524, *p*<0.001) to detect air trapping. Within the segmented lung parenchyma, AT was best defined as voxel difference less than 80 HU between expiration and registered inspiration using the DM method. DM correlated best with RV/TLC (*r* = 0.663, *p*<0.001). DM and E/I-ratio MLD showed a larger AUC (0.78; 95% CI 0.69–0.86; 0.76, 95% CI 0.67–0.85) than EXP _-790 HU to -950 HU_ (0.71, 95% CI 0.63–0.78). DM and E/I-ratio MLD showed better correlation with RV/TLC and are more suited quantitative CT-methods to detect pAT in lung transplant patients than the EXP_-790HU to -950HU_.

## Introduction

One of the most common complications and leading cause of death in patients after lung transplantation is chronic lung allograft rejection [1]. This condition, clinically known as bronchiolitis obliterans syndrome (BOS), is characterized by submucosal lymphocytic inflammation with further fibrosis of small airways, resulting in partial or complete airways obstruction [2–3]. Similar obstructive impairments of the small airways occur in COPD patients preceding parenchymal destruction (emphysema) and defined in the literature as small airways disease [4]. Pulmonary function testing (PFT) was established as standard method to assess obstructive dysfunction of lungs, however it seems less sensitive to obstructive impairments of small airways [4–6]. The presence of pathologic air trapping (AT) on expiratory CT scans has been defined as a parameter to detect small airways disease [3, 7]. On CT-scans air trapping is defined as less than normal increase of the lung parenchyma’s attenuation during expiration and a lack of volume reduction [8]. In lung transplant patients, the presence of AT on expiratory CT-scans was postulated as important biomarker to detect early chronic lung rejection [9].

During the past decade various quantitative CT AT measurements were proposed, however, CT-quantification of air trapped regions remains challenging. The most often used technique was thresholding to quantify air trapping in COPD and asthma patients using a percentage of voxels between -850 HU and -950 HU in expiration (EXP_-850 to -950_) [10–12]. Recently, Mets et al [7] compared several CT air trapping measurements in lung cancer screening participants and showed the E/I-ratio MLD is best suited for diagnosis of pathologic AT. However, a voxel-to-voxel comparative method of the inspiration and expiration CT datasets, which was suggested by Galban et al [13] for diagnosis of COPD phenotypes seems to be more accurate for assessment of small airways impairment, was not included in their study. In the present study we used density mapping (DM), a voxel-wise image measurement method based on registration of the inspiration and expiration CT datasets, which we compared with previously described techniques: E/I-ratio MLD [14–16] and the threshold-based method in expiration [10, 11]. The aim of our study was to determine, which of the three quantitative CT air trapping methods is the most suitable to quantify and to identify pathologic air trapping in patients after lung transplantation.

## Material and Methods

### Subjects

After written and informed consent and approval by the ethics committee of Hanover Medical School 155 patients were included in this study. This single-centre prospective study from January, 2009 till December, 2012, included all 150 lung and 5 heart-lung transplant recipients, who underwent base-line CT-scans 6 months after lung transplantation and body plethysmography within six hours after the CT exam. The indication for transplantation was emphysema (53 Patients), cystic fibrosis (37), pulmonary fibrosis (41), primary pulmonary hypertension (15) and COPD (9). Six patients were excluded because of inability to undergo body plethysmography within six hours after CT. Two patients with single lung transplantation were also excluded.

All transplant recipients included in the study were clinically and functionally stable and had no evidence of acute allograft rejection, acute infection or asthma after transplantation. The final number of evaluated patients with base-line CT-scans was 147.

### Spirometer controlled MDCT

Volumetric non-enhanced CT examinations were performed for all subjects at total lung capacity (TLC) and at functional residual capacity (FRC) after standardized breath-hold instructions using a spirometer as previously described [17]. All scans were performed on a 64 multi detector row CT scanner (Lightspeed VCT, GE Healthcare, Milwaukee, WI, USA) with parameters for both scans as follows: collimation 1.25 mm, a reconstruction interval of 1 mm using “standard” reconstruction kernel, with 100 kVp, automatic tube current modulation (Smart mA GE), a rotation time of 0.8 s and a pitch of 0.984.

### Pulmonary Function Testing (PFT)

Bodyplethysmography (BodyScope N, Ganshorn Medizin Electronic. GmbH, Germany) was performed within six hours after MDCT and obtained according to European Respiratory Society (ERS) guidelines [18] in 147 patients. According to the literature [7,19,20] we used the ratio of residual volume to total lung capacity (RV/TLC) as standard reference for assessment of air trapping. Air trapping was considered pathologic, if RV/TLC was above the 95th percentile of the predicted upper limit of normal values. The 95th percentile of predicted was calculated for each subject by using the formulas suggested by Stocks et al: for adult males 14.0+0.39*age+(1.64*Residual Standard Deviation (RSD)) and for adult females 19.0+0.34*age+(1.64*RSD) [20].

### CT measurements for Quantification of Air Trapping

Quantification of air trapping was performed with the threshold-based method in expiration (EXP), the density mapping method (DM), and the expiratory to inspiratory ratio of the mean lung density technique (E/I ratio). For DM, a non-rigid registration of the CT examination was performed at total lung capacity (TLC) and at functional residual capacity (FRC) prior to voxel-to-voxel mapping [21,22]. To optimize the density ranges for EXP and for DM, systematic variation of attenuation ranges was performed (see S1 Appendix).

### Data analysis

Prior to the CT measurements HU-values of the inspiration and expiration CT-scans were calibrated by measuring the HU-values of air in the tracheal lumen above the bifurcation (S1 Appendix). The correlation and agreement of the different CT measures with RV / TLC for the detection of air trapping in patients after lung transplantation as well as among quantitative CT air trapping measurements was evaluated with Spearman rank correlation test as the evaluated parameters were not normally distributed. Bland-Altman-analysis was performed for calculating the mean difference, and 95% limits of agreement between RV / TLC and each evaluated quantitative CT air trapping measures.

The receiver operating characteristic curve analysis (ROC) was performed for all evaluated quantitative CT air trapping measures for detection of pathologic AT.

For statistical analysis SPSS statistics (SPSS Inc., ver.15.0., Chicago, IL, USA), MedCalc v11.3.8.0 (Mariakerke, Belgium) and Analyse-it 2.13 (Analyse-it Software, Ltd., Leeds, UK) were used.

Values of patient demographics were expressed as mean ± SD. Values of quantitative CT air trapping measures were displayed as median and 25^th^ to 75^th^ percentile as they were not normally distributed. A P < 0.05 was considered significant.

## Results

Demographic data of all patients are illustrated in Table 1. Thirty-four (23%) patients had pathologic air trapping.

**Table 1:** Demographic data for study patients.

Patient data, n = 147	
**Males, n (%)**	87 (59%)
**Females, n (%)**	60 (41%)
**Age, year (mean ± SD** ^b^ **)**	46.6±12.5
**RV/TLC** ^a^ **, % (mean ± SD** ^b^ **)**	36.7±9.2
**Pathologic Air Trapping, n (%)**	34 (23%)

^a^ratio of residual volume to total lung capacity

^b^standard deviation

The HU-differences of tracheal air between expected (-1000 HU) and measured HU-values remained stable for the entire cohort (inspiration: 58,8 ± 9,1 HU and expiration: 69,8 ± 11,3 HU). Thus, a fixed value of 70 HU in all expiration and 60 HU in all inspiration data sets were subtracted for calibration.

### Threshold-based method in expiration

The correlation coefficients using the corrected density ranges of the expiration scan are shown in Table 2. The percentage of voxels between -790 HU to -950 HU in expiration showed the best correlation with RV/TLC (*r* = 0.52, *p*<0.001). However, varying the lower threshold between -950 to -1010 HU and the upper threshold between -770 HU to -810 HU revealed no significant correlation improvement (P>0.05).

**Table 2:** Spearman´s correlation coefficients (r) of the threshold-based method in expiration for varying threshold ranges.

Lower threshold Upper threshold	-930 HU	-950 HU	-970 HU	-990 HU	-1010HU
**-870HU**	0.482	0.501	0.499	0.489	0.470
**-850 HU**	0.495	0.512	0.506	0.499	0.476
**-830 HU**	0.498	0.514	0.510	0.506	0.480
**-810 HU**	0.506	0.522	0.516	0.514	0.487
**-790 HU**	0.505	**0.524**	0.519	0.517	0.495
**-770 HU**	0.493	0.518	0.515	0.510	0.475
**-750 HU**	0.478	0.489	0.480	0.460	0.457

The percentage of voxels between -790 HU to -950 HU showed the best correlation with RV/TLC (r = 0.52, p<0.001). However, varying the lower threshold between (-950 to -1010 HU) and the upper threshold between (-770 HU to -810 HU) revealed no significant correlation improvement (P>0.05).

### Density mapping

The correlation of detected AT in the DM with RV/TLC is shown in Table 3.

**Table 3:** Spearman´s correlation coefficients (r) of detected AT in the DM with RV/TLC for varying attenuation ranges and varying HU-differences between registered inspiration and expiration.

HU-difference Density Ranges	±50 HU	±60 HU	±70 HU	±80 HU	±90 HU
**-700 to -950 HU**	0.633	0.633	0.635	0.635	0.627
**-600 to -950 HU**	0.639	0.645	0.647	0.646	0.645
**-500 to -950 HU**	0.646	0.651	0.655	0.653	0.662
**-400 to -950 HU**	0.649	0.657	0.662	**0.663**	0.659
**-300 to -950 HU**	0.651	0.657	0.663	**0.663**	0.662
**-200 to -950 HU**	0.651	0.657	0.663	**0.663**	0.662
**-100 to -950 HU**	0.651	0.657	0.663	**0.663**	0.662
**0 to -950 HU**	0.651	0.657	0.663	**0.663**	0.662

DM was robust regarding the attenuation ranges of the upper threshold and the HU-differences showing similar correlation of DM with RV/TLC for all parameter settings (0.62 ≤ r ≤ 0.66).

The attenuation ranges with upper threshold from -400 HU to 0 and lower threshold of -950 HU in expiration with the HU-difference (DI) of ±80 HU between registered inspiration and expiration showed the best correlation with RV/TLC (*r* = 0.66, *p*<0.001). However, DM was quite robust regarding the attenuation value of the upper threshold and the HU-differences showing similar correlation of DM with RV/TLC for all parameter settings (0.62 ≤ r ≤ 0.66).

### E/I-ratio MLD

The correlation coefficient between E/I-ratio MLD and RV/TLC was 0.58 (*p*<0.001).

The correlations of the quantitative CT AT measures and RV/TLC are illustrated in Table 4. The results show that RV/TLC best correlated with DM. DM significantly correlated with E/I-ratio MLD (r = 0.90; *p*<0.001) and EXP _-790 HU to -950 HU_ (r = 0.80; *p*<0.001). E/I-ratio MLD correlated with EXP _-790 HU to -950 HU_ (r = 0.87; *p*<0.001).

**Table 4:** Spearman rank correlation´s coefficients between the quantitative CT AT measures and RV/TLC.

CT AT measures PFT (RV/TLC)	DM	E/I-ratio MLD	EXP _-790 HU to -950 HU_
**RV/TLC**	0.66	0.58	0.52

### Bland-Altman Analysis

**Fig 1:**
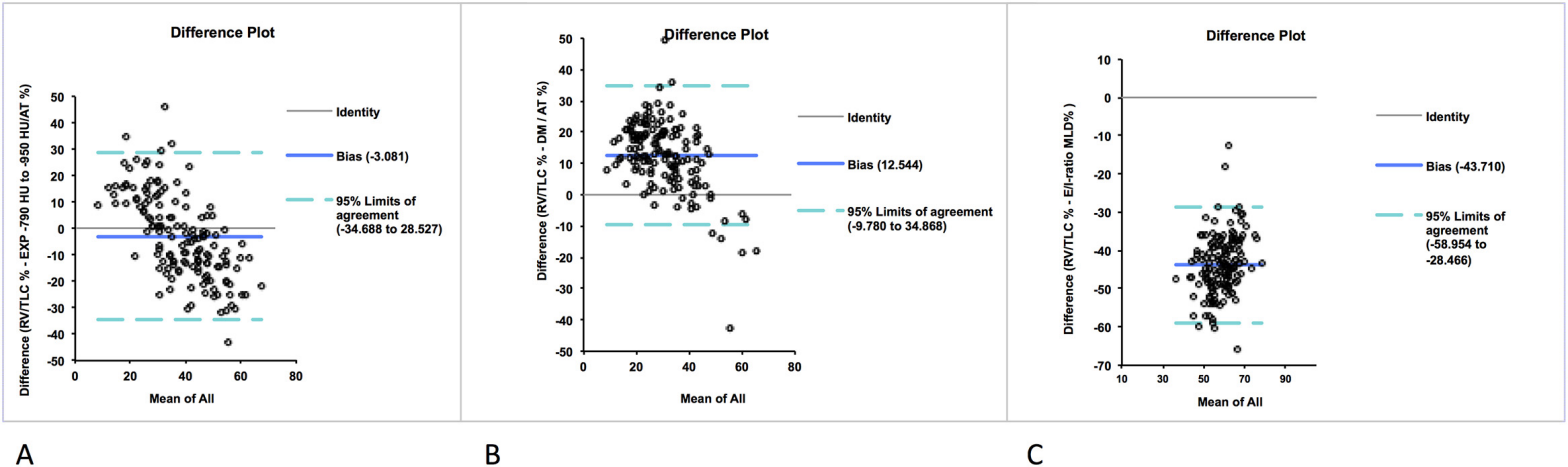
Bland-Altman plots of RV/TLC against the three evaluated CT measures for quantification of AT%. In comparison to RV / TLC, EXP_-790 to -950 HU_ (A) showed the smallest mean difference (bias) of -3.08% but the highest range in the 95% limits of agreement (-34.7% to 28.5%). E/I-ratio MLD and DM (B, C) showed smaller ranges of the 95% limits of agreement (-58.9% to -28.5% and -9.8% to 34.8%, respectively) suggesting a higher agreement with RV / TLC. Bias was highest with E/I-ratio MLD (-43.7%) while DM showed a moderate bias of 12.5%.

Bland-Altman analysis for quantification of AT% showed a bias for E/I-ratio MLD (-43.7%, p<0.001) and for DM (12.5%, p<0.001) against RV/TLC. The lowest bias was found for EXP _-790 HU to -950 HU_. (-3.1%, p = 0.02). After correction for bias, the 95% limits of agreement between EXP _-790 HU to -950 HU_ and RV/TLC were ±31.6%. The limits of agreement between DM and RV/TLC were ± 22.3%, and between E/I-ratio MLD and RV /TLC ± 15.2%. (Fig 1).

### Analysis of the ROC curves

**Fig 2:**
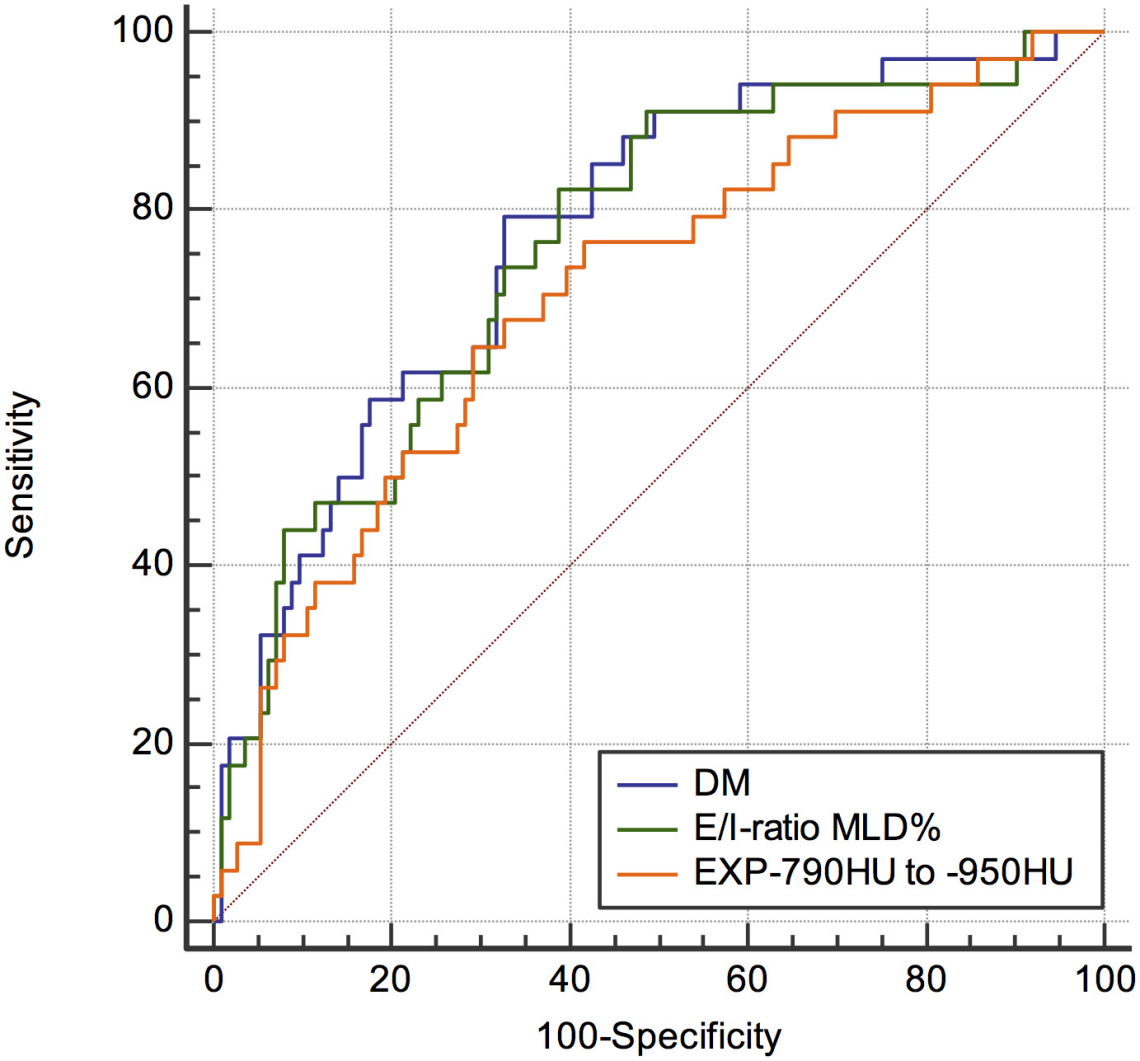
ROC curves of three quantitative CT measures to detect pAT in patients after lung transplantation. Measurements were corrected for bias. Density mapping showed the largest AUC under the ROC curve of 0.78.

ROC analysis of the three CT methods after correction for the bias (Fig 2) showed similar area under the ROC curve (AUC) by two methods: DM of 0.78 (95% CI 0.691–0.865, *p*<0.001) and E/I-ratio MLD (0.76, 95% CI 0.669–0.852, *p*<0.001); the AUC by EXP _-790 HU to -950 HU_ was significantly lower (0.71, 95% CI 0.606–0.807, *p*<0.001). Pairwise comparison of ROC curves of DM and E/I-ratio showed no significant difference (P = 0.63), whereas the ROC curves between DM and EXP _-790 HU to -950 HU_ (P = 0.011) as well as E/I-ratio and EXP _-790 HU to -950 HU_ (P = 0.011) were significantly different.

## Discussion

In our study we compared three quantitative CT air trapping’s measures for quantification and detection of pathologic air trapping in patients after lung transplantation. We showed that DM and E/I-ratio MLD are more suitable quantitative CT air trapping methods in lung transplant patients than the thresholding technique. An advantage of DM is its ability to evaluate regional distribution of AT as voxel-to-voxel mapping. In contrast, E/I-ratio MLD only provides a global measure of AT.

Air trapping is accepted as an indirect sign for small airway remodelling and is present in various obstructive airways diseases, such as COPD [23], asthma [12], cystic fibrosis [24], and atypical pneumonia [25]. PFTs are not sensitive enough for the detection of small airway impairment. The necessity of expiratory CT scans for diagnosis of air trapping was confirmed in many studies [9, 26, 27]. The attenuation range in expiration from -850 HU to -950 HU was published as the most applicable to identify air trapping in COPD and asthma patients excluding emphysema [10–12]. In the case of lung transplant patients both transbronchial biopsy and PFTs are insensitive for early recognition of the bronchiolitis obliterans syndrome (BOS), while the presence of air trapping on expiratory CT scans was suggested as the most suitable sign for diagnosis [9, 27, 28]. According to our knowledge there were only few studies about air trapping quantification in patients after lung transplantation [9, 27, 28]. They aimed mostly at the application of expiratory CT scans for diagnosis of air trapping and did not suggest any fixed attenuation ranges. In the present work we studied different attenuation ranges for the threshold-based method in expiration to quantify air trapping in patients after lung transplantation and showed that the range from -950 HU to -790 HU after tracheal correction as the best correlated with RV/TLC. Our result differs from previously published data in COPD and asthma patients [10–12] regarding the upper threshold. The higher upper threshold (up to -790 HU) in the present study may be due to deeper expiration in lung transplant patients compared to patients with COPD or asthma. Recently Mets et al [7] compared three quantitative CT air trapping methods: E/I-ratio MLD, percentage of voxels less than −856 HU in expiration (EXP_-856 HU_), and expiratory to inspiratory relative volume change of voxels with attenuation values between −860 and −950 Hounsfield Units (RVC _-860 to -950 HU_) on a population of current and former heavy smokers in a lung cancer screening setting and identified the E/I-ratio MLD as the most suited measurement to detect pathologic air trapping.

Other studies suggested the usefulness of the full 3D co-registration of inspiration and expiration CT datasets for the quantification and identification of small airway remodelling [13, 21, 29]. Galban et al [13] evaluated the parametric response map (PRM) as a successful imaging biomarker for the differentiation of two main COPD phenotypes: functional small airways disease and emphysema. In our study we evaluated a similar method for detection of pathologic air trapping in patients after lung transplantation, which differs from the Galban method of AT detection: Galban et al [13] used fixed threshold ranges in inspiration as well as in expiration for quantification of AT areas. In the DM method presented in this paper, only one threshold range in expiration was set to define the target area including the ventilated lung parenchyma and excluding emphysema, vessels, and interstitial tissue. To separate AT areas from healthy lung parenchyma, a maximum HU-difference of 80 HU between voxels in the registered inspiration and the expiration was implemented as the main restriction. We found that the best attenuation ranges on scatter plots for DM were the following: upper threshold between -400 HU to 0 and lower threshold of -950 HU; the most appropriate HU-difference (DI) between inspiration and expiration was ±80 HU (*r* = 0.663, *p*<0.001). DM was quite robust and results were only mildly dependent on parameter settings. The method of density mapping for the assessment of AT included both a broad threshold range to select the target area and a restriction of HU differences in the inspiration and expiration data. The higher sensitivity (broadening of the threshold range), and the higher specificity (HU difference limit between inspiration and expiration), outperformed the established threshold method in expiration for the detection of pathologic air trapping.

In comparison to DM, E/I-ratio MLD performed similarly in the ROC, in the Bland-Altman analysis and in the Spearman correlation rank test. DM tended to underestimate air trapping, as it measured AT more specific than RV/TLC, excluding emphysematous regions. On the contrary, E/I-ratio MLD overestimated AT. This might be due to too high density values of the lung parenchyma in the expiration scan caused by deficiencies of current reconstruction algorithms for volumetric CT [30]. We performed density correction using measurements of tracheal air as mentioned in the manuscript. However, a full correction of density values could not be achieved. E/I-ratio MLD was simpler to calculate without the time consuming and costly registration. However, density mapping may allow for additional assessment of AT on the regional level, while E/I-ratio MLD is only a global lung AT parameter. Future investigations of density mapping are required to evaluate regional assessment of lung function, which could considerably help to detect early chronic lung rejection and complement routine lung function tests.

This study has several limitations. First, there was no direct measure of AT available as pathologic evidence. The single-breach nitrogen washout test has been suggested [31, 32] as an excellent tool for precise assessment of the structural changes of small airways. However, using this method was not possible because of limited access to this equipment during our study. The RV/TLC was used as a very good measurement of functional changes of airways [20] and due to its wide availability. Though, it is not sufficiently sensitive to mild obstructive abnormalities of small airways [4–6]. In addition, the normality of air trapping is still open to question and its severity may be influenced by age in normal population as well [33]. The definition of pathologic AT in our study was based on the formula suggested by Stocks et al [20]: RV/TLC above the 95th percentile of the predicted upper limit of normal values, which was dependent on sex and age of the patients. However, this formula has not been validated for lung-transplant patients. Second, the non-rigid registration technique had some limitations. Although substantial misalignments were not present, it can be assumed that the peripheral regions of the lung were prone to distortions. Therefore, a potential registration error may be present. Another potential limitation of the study may be the lack of assessment of lobe-based air trapping. The DM method correlated only slightly better with RV/TLC than the other two measurements. Future evaluation of DM method after lobe segmentation may help to optimize its upper threshold and the HU-difference and could improve the utility of DM method.

In conclusion, both density mapping and E/I-ratio MLD are better suited to detect pathologic air trapping in patients after lung transplantation than the threshold method. In addition, DM can provide information about the regional distribution of AT.

## Supporting Information

S1 Appendix (DOC)

S1 Fig Evaluation of the mask for the lung parenchyma in inspiration and expiration. (TIFF)

S2 Fig Frequency distribution of coincided voxels of the expiration (y-axis) and registered inspiration (x-axis) data. (TIFF)

S3 Fig Schematic diagram of the density mapping method. (TIFF)

## References

[1] Arcasoy SM, Kotloff RM. Lung transplantation. N Engl J Med. 1999;340(14):1081–91. 10194239 10.1056/NEJM199904083401406

[2] Boehler A, Estenne M. Obliterative bronchiolitis after lung transplantation. Curr Opin Pulm Med. 2000;6(2):133–9. 10741773 10.1097/00063198-200003000-00009

[3] Kauczor HU, Wielpütz MO, Owsijewitsch M, Ley-Zaporozhan J. Computed tomographic imaging of the airways in COPD and asthma. J Thorac Imaging. 2011;26(4):290–300. 10.1097/RTI.0b013e3182277113 22009082

[4] Friedlander AL, Lynch D, Dyar LA, Bowler RP. Phenotypes of chronic obstructive pulmonary disease. COPD. 2007;4(4):355–384. 18027163 10.1080/15412550701629663

[5] Global Initiative for Chronic Obstructive Pulmonary Disease. Global strategy for the diagnosis, management, and prevention of chronic obstructive pulmonary disease. Updated 2010. Available at: http://www.goldcopd.com. Accessed September 2011.

[6] Reilly JJ. COPD and declining FEV1—time to divide and conquer? N Engl J Med. 2008;359(15):1616–1618. 10.1056/NEJMe0807387 18836214

[7] Mets OM, Zanen P, Lammers JW, Isgum I, Gietema HA, van Ginneken B et al Early Identification of Small Airways Disease on Lung Cancer Screening CT: Comparison of Current Air Trapping Measures. Lung. 2012 12;190(6):629–33. 10.1007/s00408-012-9422-8 23064488

[8] Hansell DM, Bankier AA, MacMahon McLoud TC, Muller NL, Remy J. 2008 Fleischner Society: glossary of terms for thoracic imaging. Radiology. 246, 3, 697–722. 10.1148/radiol.2462070712 18195376

[9] Bankier AA, Van Muylem A, Knoop C, Estenne M, Gevenois PA. Bronchiolitis obliterans syndrome in heart-lung transplant recipients: diagnosis with expiratory CT. Radiology. 2001 2;218(2):533–9. 11161175 10.1148/radiology.218.2.r01fe09533

[10] Gevenois PA, Vuyst P, Sy M, Scillia P, Chaminade L, Maertelaer V et al Pulmonary emphysema: quantitative CT during expiration. Radiology. 1996;199(3):825–829. 8638012 10.1148/radiology.199.3.8638012

[11] van Ginneken B, Murphy K, van Rikxoort EM, Isgum I, de Hoop B, Prokop M et al. (2009) Quantification of Emphysema and Small Airway Disease in COPD Patients from Lobar Analysis of Volumetric Inspiration and Expiration Thoracic CT Scans. Radiological Society of North America, 95th Annual Meeting.

[12] Busacker A, Newell JD Jr, Keefe T, Hoffman EA, Granroth JC, Castro M et al A multivariate analysis of risk factors for the air-trapping asthmatic phenotype as measured by quantitative CT analysis. Chest. 2009;135(1):48–56. 10.1378/chest.08-0049 18689585 PMC2849984

[13] Galbán CJ, Han MK, Boes JL, Chughtai KA, Meyer CR, Johnson TD et al Computed tomography-based biomarker provides unique signature for diagnosis of COPD phenotypes and disease progression. Nat Med. 2012 11;18(11):1711–5. 10.1038/nm.2971 23042237 PMC3493851

[14] Kubo K, Eda S, Yamamoto H, Fujimoto K, Matsuzawa Y, Maruyama Y, Hasegawa M et al Expiratory and inspiratory chest computed tomography and pulmonary function tests in cigarette smokers. Eur Respir J. 1999;13(2):252–256. 10065664 10.1034/j.1399-3003.1999.13b06.x

[15] Eda S, Kubo K, Fujimoto K, Matsuzawa Y, Sekiguchi M, Sakai F. The relations between expiratory chest CT using helical CT and pulmonary function tests in emphysema. Am J Respir Crit Care Med. 1997;155(4):1290–1294. 9105069 10.1164/ajrccm.155.4.9105069

[16] O'Donnell RA, Peebles C, Ward JA, Daraker A, Angco G, Broberg P et al Relationship between peripheral airway dysfunction, airway obstruction, and neutrophilic inflammation in COPD. Thorax. 2004;59(10):837–842. 15454648 10.1136/thx.2003.019349 PMC1746844

[17] Dettmer S, Peters L, de Wall C, Schaefer-Prokop C, Schmidt M, Warnecke G et al Bronchial wall measurements in patients after lung transplantation: evaluation of the diagnostic value for the diagnosis of bronchiolitis obliterans syndrome. PLoS One. 2014; 9(4): e93783 10.1371/journal.pone.0093783 24713820 PMC3979715

[18] Miller MR1, Crapo R, Hankinson J, Brusasco V, Burgos F, Casaburi R et al General considerations for lung function testing. Eur Respir J. 2005;26:153–161. 15994402 10.1183/09031936.05.00034505

[19] Quanjer PH, Tammeling GJ, Cotes JE, Pedersen OF, Peslin R, Yernault JC. Report Working Party Standardization of Lung Function Tests, European Community for Steel and Coal. Official Statement of the European Respiratory Society. Eur Respir J Suppl. 1993;16:5–40. 8499054

[20] Stocks J, Quanjer PH. Reference values for residual volume, functional residual capacity and total lung capacity. ATS Workshop on Lung Volume Measurements. Official Statement of The European Respiratory Society. Eur Respir J. 1995;8(3):492–506. 7789503 10.1183/09031936.95.08030492

[21] Murphy K, Pluim JP, van Rikxoort EM, de Jong PA, de Hoop B, Gietema HA et al Toward automatic regional analysis of pulmonary function using inspiration and expiration thoracic CT. Med Phys. 2012; 39(3):1650–62. 10.1118/1.3687891 22380397

[22] Avants BB, Tustison NJ, Song G, Cook PA, Klein A, Gee JC. A reproducible evaluation of ANTs similarity metric performance in brain image registration. Neuroimage. 2011; 54(3):2033–44. 10.1016/j.neuroimage.2010.09.025 20851191 PMC3065962

[23] Matsuoka S, Kurihara Y, Yagihashi K, Hoshino M, Watanabe N, Nakajima Y. Quantitative assessment of air trapping in chronic obstructive pulmonary disease using inspiratory and expiratory volumetric MDCT. AJR Am J Roentgenol. 2008;190(3):762–9. 10.2214/AJR.07.2820 18287450

[24] Goris ML, Zhu HJ, Blankenberg F, Chan F, Robinson TE. An automated approach to quantitative air trapping measurements in mild cystic fibrosis. Chest. 2003;123(5):1655–63. 12740287 10.1378/chest.123.5.1655

[25] Kubo K, Yamazaki Y, Masubuchi T, Takamizawa A, Yamamoto H, Koizumi T et al Pulmonary infection with Mycobacterium avium-intracellulare leads to air trapping distal to the small airways. Am J Respir Crit Care Med. 1998;158(3):979–84. 9731034 10.1164/ajrccm.158.3.9802042

[26] Arakawa H, Webb WR. Air trapping on expiratory high-resolution CT scans in the absence of inspiratory scan abnormalities: correlation with pulmonary function tests and differential diagnosis. AJR Am J Roentgenol. 1998;170(5):1349–53. 9574614 10.2214/ajr.170.5.9574614

[27] Leung AN, Fisher K, Valentine V, Girgis RE, Berry GJ, Robbins RC et al Bronchiolitis obliterans after lung transplantation: detection using expiratory HRCT. Chest. 1998;113(2):365–70. 9498953 10.1378/chest.113.2.365

[28] Siegel MJ, Bhalla S, Gutierrez FR, Hildebolt C, Sweet S. Post-lung transplantation bronchiolitis obliterans syndrome: usefulness of expiratory thin-section CT for diagnosis. Radiology 2001;220(2):455–62. 11477251 10.1148/radiology.220.2.r01au19455

[29] Murphy K, van Ginneken B, Reinhardt JM, Kabus S, Ding K, Deng X et al Evaluation of methods for pulmonary image registration: The EMPIRE10 study, Medical Image Analysis for the Clinic: A Grand Challenge (2010), pp. 11–22

[30] Kim SS, Seo JB, Kim N, Chae EJ, Lee YK, Oh YM et al Improved correlation between CT emphysema quantification and pulmonary function test by density correction of volumetric CT data based on air and aortic density. Eur J Radiol. 2014 1; 83(1):57–63. 10.1016/j.ejrad.2012.02.021 22613510

[31] Bourdin A, Kotsimbos T, Nguyen K, Vachier I, Mainprice B, Farce M et al Non-invasive assessment of small airway remodelling in smokers. COPD 2010, 7(2):102–110. 10.3109/15412551003631709 20397810

[32] Bommart S, Marin G, Bourdin A, Molinari N, Klein F, Hayot M et al Relationship between CT air trapping criteria and lung function in small airway impairment quantification. BMC Pulm Med. 2014 2 28;14:29 10.1186/1471-2466-14-29 24581147 PMC4015710

[33] Bommart S, Marin G, Bourdin A, Revel MP, Klein F, Hayot M et al Computed tomography quantification of airway remodelling in normal ageing subjects: a cross-sectional study. Eur Respir J. 2015 4;45(4):1167–70. 10.1183/09031936.00215314 25537558

