# Supplementary material for: Quantification of Pathologic Air Trapping in Lung Transplant Patients Using CT Density Mapping: Comparison with Other CT Air Trapping Measures
Source: PLoS One. 2015 Oct 2;10(10):e0139102. doi: 10.1371/journal.pone.0139102 (PMC4592198; doi:10.1371/journal.pone.0139102)
Supplement: S1 Appendix — (DOC) [file pone.0139102.s001.doc]

**S1 Appendix**

**Tracheal correction of attenuation values**

Prior to the CT measurements HU-values of the inspiration and expiration CT-scans were calibrated by measuring the HU-values of air in the tracheal lumen above the bifurcation. A ROI of at least 1 cm^2^ was applied to calculate the difference between expected (-1024 HU) and measured value of air in the tracheal lumen. The calculated difference was used for calibration of the HU-values.

**Lung segmentation**

The segmentation of the lung parenchyma was performed for all three quantitative CT air-trapping measures. For the threshold-based method in expiration and the density mapping the lung segmentation consisted of the following steps: a mask for both lungs was automatically generated by applying a local, adaptive region growing algorithm in inspiration and expiration; the resulting mask was overlain on the original data in order to exclude all other tissue except for the lung parenchyma in inspiration and expiration; Mevislab (MeVisLab SDK 2.2.1, MeVis Medical Solutions AG, Bremen, Germany) (S1 Fig).

The lung segmentation for the E/I-ratio MLD [14-16] was performed using dedicated software (MeVisPULMO 3D, Fraunhofer MEVIS Bremen, Germany).

**S1 Fig. Evaluation of the mask for the lung parenchyma in inspiration and expiration.** A mask for both lungs was automatically generated using a threshold-based, locally adaptive region-growing algorithm. The resulting mask was overlain on the original data in order to extract the lung parenchyma in inspiration and expiration.

**Threshold-based method in expiration**

The established HU-range of EXP_-850 to -950_ [10-12] was varied systematically in both directions with increments of -20 HU (lower threshold between -950 to -1010 HU and the upper threshold between -750 HU to -810 HU). The segmented volume of all density ranges in relation to the total lung volume was correlated to the ratio of residual volume to total lung capacity (RV/TLC) by Spearman rank correlation tests. The threshold range with the highest correlation was considered the most suitable density range in expiration for AT quantification in lung transplant patients.

**Density mapping**

For density mapping the following steps were performed:

1. To achieve a voxel-to-voxel correspondence of the HU values in inspiration and expiration, the inspiration CT scan was spatially deformed to match the expiration CT scan. This spatial deformation was performed with non-rigid registration using an open-source software package (ANTS, 2011, Release 1.5, Penn Image Computing and Science Laboratory, University of Pennsylvania, USA) and included the following steps: first, an affine transformation was applied to correct for translation, rotation, scaling and shearing differences in in- and expiration; second, a non-rigid registration was performed using a diffeomorphic transformation model and cross correlation as the similarity function. For both, the affine and the diffeomorphic registration, the gradient descent was used for the optimization as described elsewhere [21, 22].

2. The frequency distribution of the coincided voxels of the expiration (y-axis) and the registered inspiration (x-axis) data was depicted on a scatter plot using a logarithmically scaled color coding (S2 Fig).

3. The bisector line represented no change in HU-values between the registered inspiration and the expiration. By specifying an upper and lower threshold value in the expiration data and a maximum allowable HU-difference between the expiration and the registered inspiration data, a bounding parallelogram was defined. Its area enclosed all corresponding density pairs in the registered inspiration and expiration data assigned to air trapping (S2 Fig). A systematic variation of density ranges on the scatter plot was performed to optimize the position and the size of the parallelogram using a fixed lower threshold of -950 HU, while the upper threshold was systematically varied from -700 HU to 0 with a step size of 100 HU in the expiration data. The maximum allowable HU-difference between the expiration and the registered inspiration data was varied from -50 to -90 HU with an increment of 10 HU.

To find out the best density range for the density mappings, Spearman’s correlation coefficients were obtained comparing RV/TLC with varying attenuation ranges in all patients. The number of the voxel pairs included in the parallelogram in relation to the total lung volume gave the relative volume of AT for comparison with RV/TLC. A flow chart outlining the density mapping is shown in S3 Fig.

**S2 Fig.** **Frequency distribution of coincided voxels of the expiration (y-axis) and registered inspiration (x-axis) data.** The bisector line represents no change in HU-values between the registered inspiration and the expiration. The area within the parallelogram included all voxel pairs with HU values in the range of -400 and -950 HU in expiration and a maximum difference between inspiration and expiration of 80 HU in this example. All voxel pairs corresponding to areas with air trapping are within the borders of the parallelogram.

**S3 Fig.** **Schematic diagram of the density mapping method.** CT-data of inspiration and expiration were automatically segmented. Following segmentation, the lungs in inspiration were non-rigidly registered to the lungs in expiration. The resulting voxel-to-voxel correspondence of the registered inspiration CT dataset to the expiration CT dataset allowed for the analysis of the frequency distribution of the matching voxels in expiration and the registered inspiration using a scatter plot. The detected AT regions with DM were overlain on the expiration scan showing the regional distribution. The color-coded areas in blue correspond to the voxels enclosed within the parallelogram depicting the regional distribution of air trapping.

**E/I-ratio MLD**

The E/I-ratio MLD [14-16] was automatically calculated using proprietary software (MeVisPULMO 3D, Fraunhofer MEVIS Bremen, Germany). Following automatic lung segmentation, mean lung densities in inspiration and expiration as well as their ratio were computed and correlated to RV / TLC.
