# Supplementary figures and images for: Quantification of Pathologic Air Trapping in Lung Transplant Patients Using CT Density Mapping: Comparison with Other CT Air Trapping Measures
Source: PLoS One. 2015 Oct 2;10(10):e0139102. doi: 10.1371/journal.pone.0139102 (PMC4592198; doi:10.1371/journal.pone.0139102)

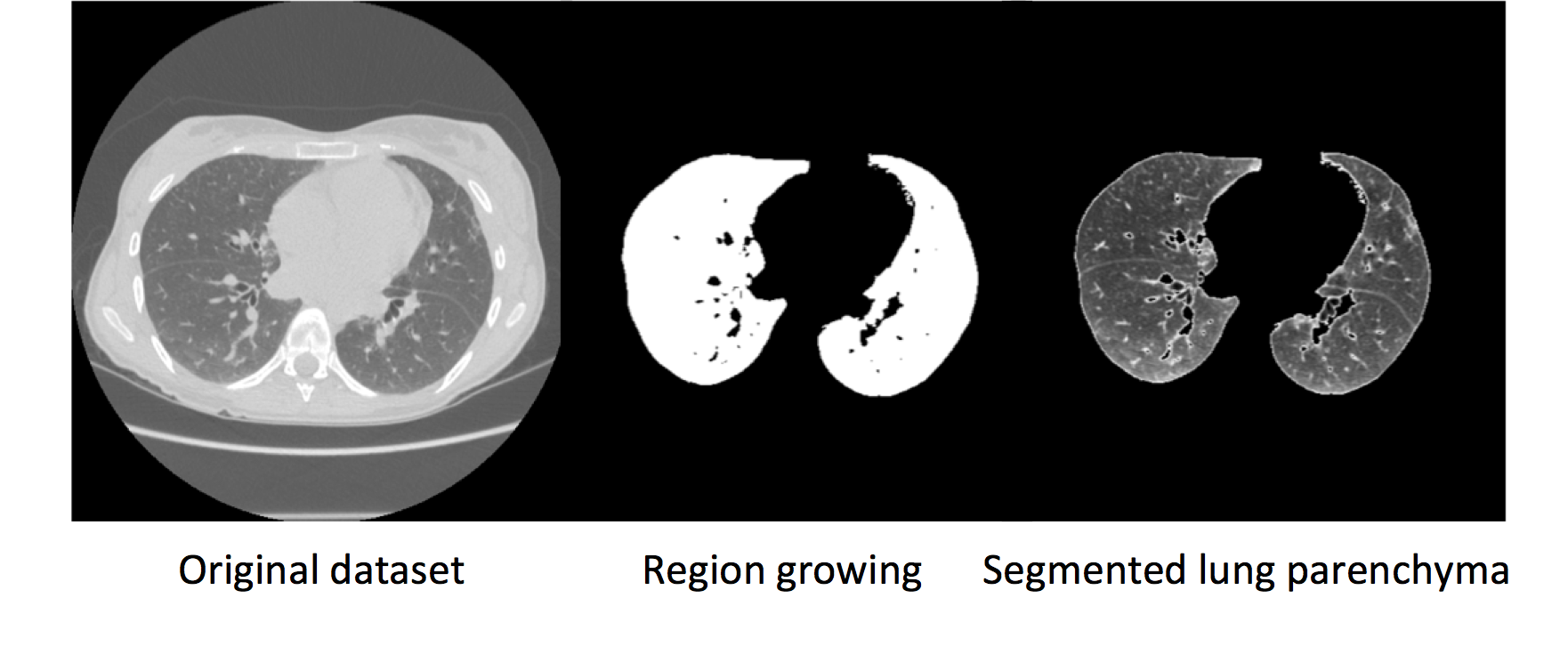

Supplement: S1 Fig — (TIFF) [file pone.0139102.s002.tiff]

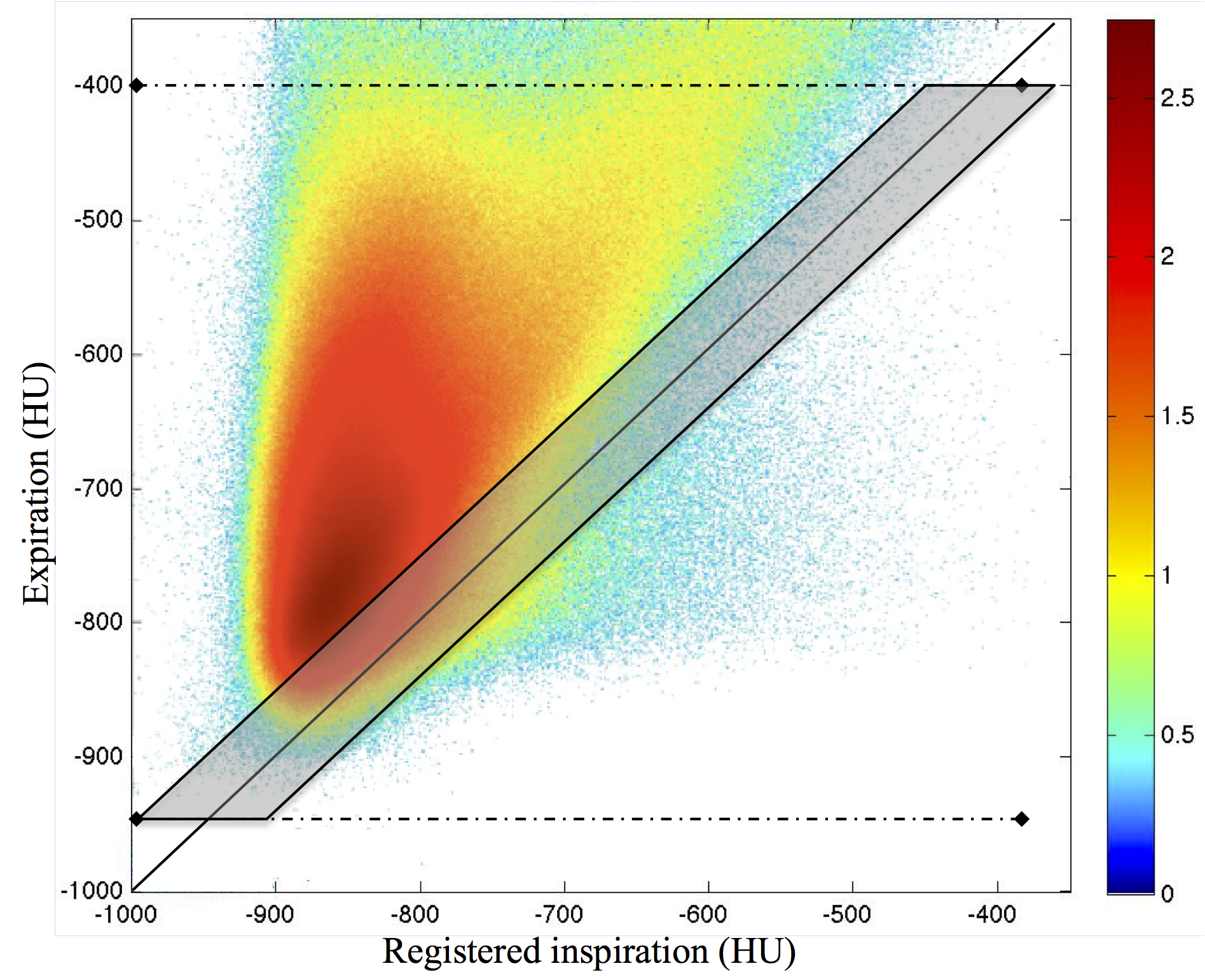

Supplement: S2 Fig — (TIFF) [file pone.0139102.s003.tiff]

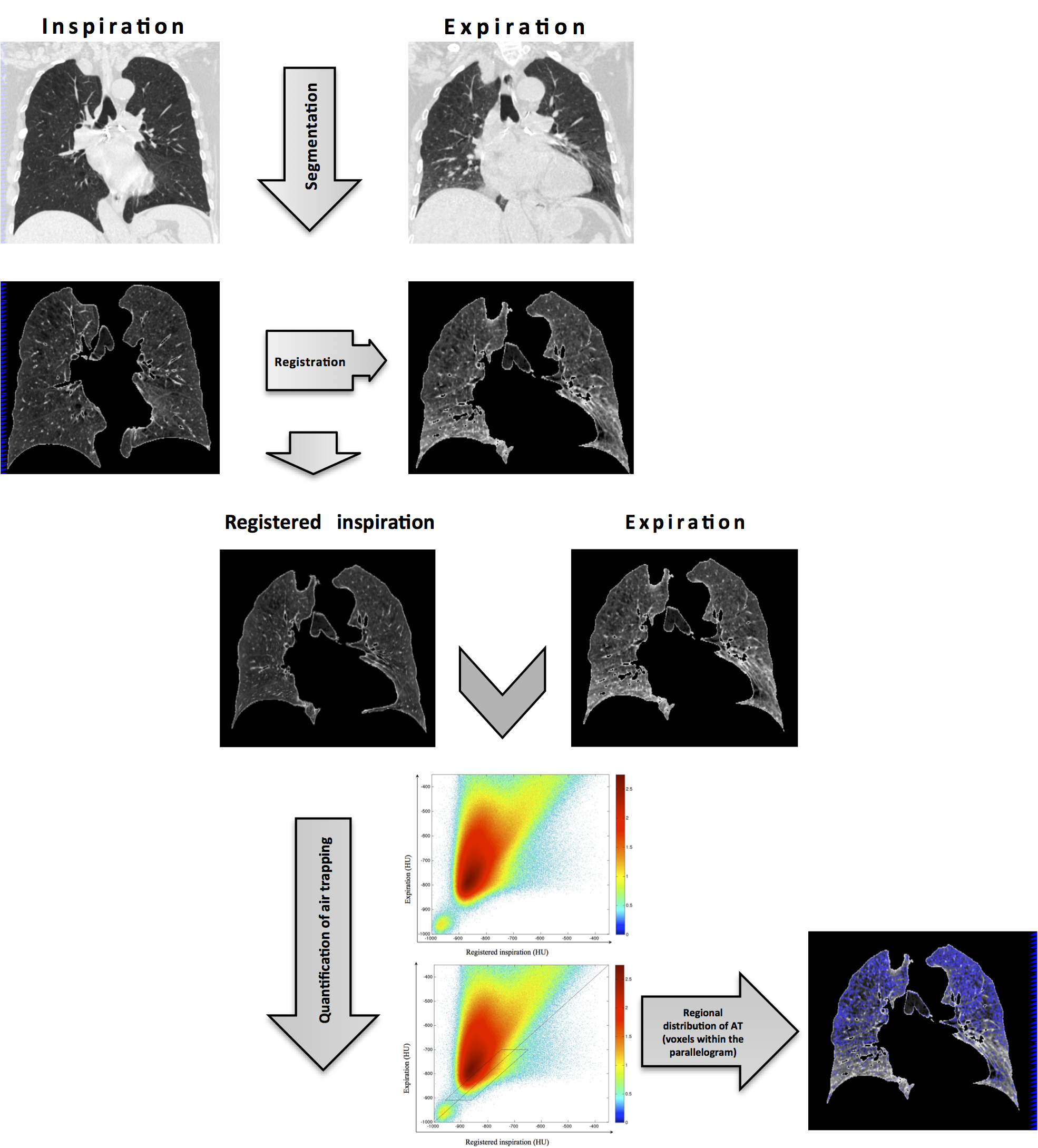

Supplement: S3 Fig — (TIFF) [file pone.0139102.s004.tiff]
